# α_1_ Proline 277 Residues Regulate GABA_A_R Gating through M2-M3 Loop Interaction in the Interface Region

**DOI:** 10.1021/acschemneuro.2c00401

**Published:** 2022-10-11

**Authors:** Przemyslaw T. Kaczor, Michał A. Michałowski, Jerzy W. Mozrzymas

**Affiliations:** Department of Biophysics and Neuroscience, Wroclaw Medical University, Chalubinskiego 3a, Wroclaw, Dolnoslaskie50-368, Poland

**Keywords:** structure−function relationship, Cys-loop receptor, M2-M3 loop, gating, patch clamp, interface interaction

## Abstract

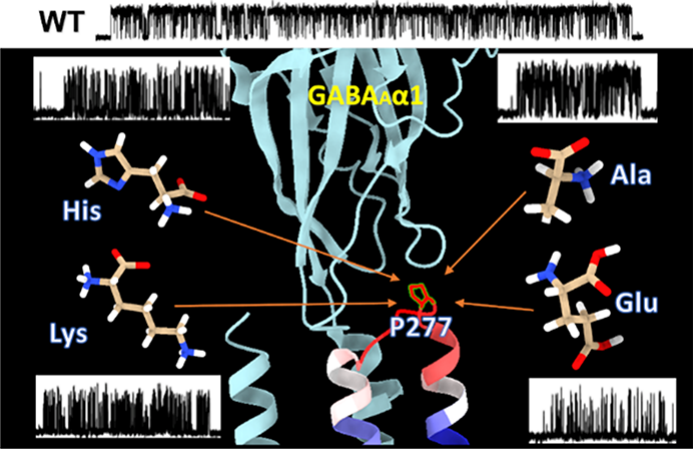

Cys-loop receptors are a superfamily of transmembrane, pentameric receptors that play a crucial role in mammalian CNS signaling. Physiological activation of these receptors is typically initiated by neurotransmitter binding to the orthosteric binding site, located at the extracellular domain (ECD), which leads to the opening of the channel pore (gate) at the transmembrane domain (TMD). Whereas considerable knowledge on molecular mechanisms of Cys-loop receptor activation was gathered for the acetylcholine receptor, little is known with this respect about the GABA_A_ receptor (GABA_A_R), which mediates cellular inhibition. Importantly, several static structures of GABA_A_R were recently described, paving the way to more in-depth molecular functional studies. Moreover, it has been pointed out that the TMD-ECD interface region plays a crucial role in transduction of conformational changes from the ligand binding site to the channel gate. One of the interface structures implicated in this transduction process is the M2-M3 loop with a highly conserved proline (P277) residue. To address this issue specifically for α_1_β_2_γ_2L_ GABA_A_R, we choose to substitute proline α_1_P277 with amino acids with different physicochemical features such as electrostatic charge or their ability to change the loop flexibility. To address the functional impact of these mutations, we performed macroscopic and single-channel patch-clamp analyses together with modeling. Our findings revealed that mutation of α_1_P277 weakly affected agonist binding but was critical for all transitions of GABA_A_R gating: opening/closing, preactivation, and desensitization. In conclusion, we provide evidence that conservative α_1_P277 at the interface is strongly involved in regulating the receptor gating.

## Introduction

Cys-loop receptors are a superfamily of transmembrane pentameric receptors that are responsible in mammalian CNS for excitatory or inhibitory transmission.^1^ Most extensively investigated representatives of this superfamily are nicotinic acetylcholine (nACh), serotonin (5-HT3), glycine (GlyR), and GABA_A_ receptors. Cys-loop receptors have transmitter binding sites located on the extracellular domain (ECD), which are particularly distant (around 50 Å) from the channel gate situated roughly in the middle of the pore in the transmembrane domain (TMD).^2^ Thus, activation of the Cys-loop receptors is believed to occur as a consequence of a complex “wave” of structural changes within the receptor macromolecule that starts upon ligand binding and eventually leads to channel pore opening.^3−9^ The activation process of the Cys-loop receptor was best described for the nicotinic acetylcholine receptors.^10−16^ Much less is known about anion-selective GABA_A_ receptors, which in an adult mammalian brain mediate inhibition.^17^ Importantly, GABA_A_Rs are targets for many clinically important pharmacological agents such as benzodiazepines, barbiturates, or anesthetics.^18−22^ Several studies dedicated to molecular mechanisms of GABA_A_R activation have shown that mutation of residues located near the GABA binding site affected not only the binding step but also channel gating.^23−27^ These results suggest that the mechanism of the GABA_A_R activation involving energy transfer from the binding side to the channel gate might occur in a form of the widespread structural rearrangements as it was proposed for nAChRs.^6^ Such a mechanism of energy transfer from the ECD to the TMD in GABA_A_Rs would require long-range interactions comprising probably most of the receptor macromolecule structures as it was proposed for nAChRs.^15^ It has been shown that the interface region between the ECD and the TMD plays a crucial role in the activation process of the pentameric ligand-gated ion channels.^7,14,28−30^ A key role of this interface has been also reported for GABA_A_Rs, where mutations introduced to this region strongly affected the receptor function in a manner suggesting an impact on receptor gating, although these effects were not characterized in detail.^8,31−34^ Moreover, recently reported high-resolution structures revealed that the interface region of GABA_A_R plays an important role in signal transduction and activation^35,36^ and overall shaping of GABA_A_R kinetics.^37^

**Figure 1:**
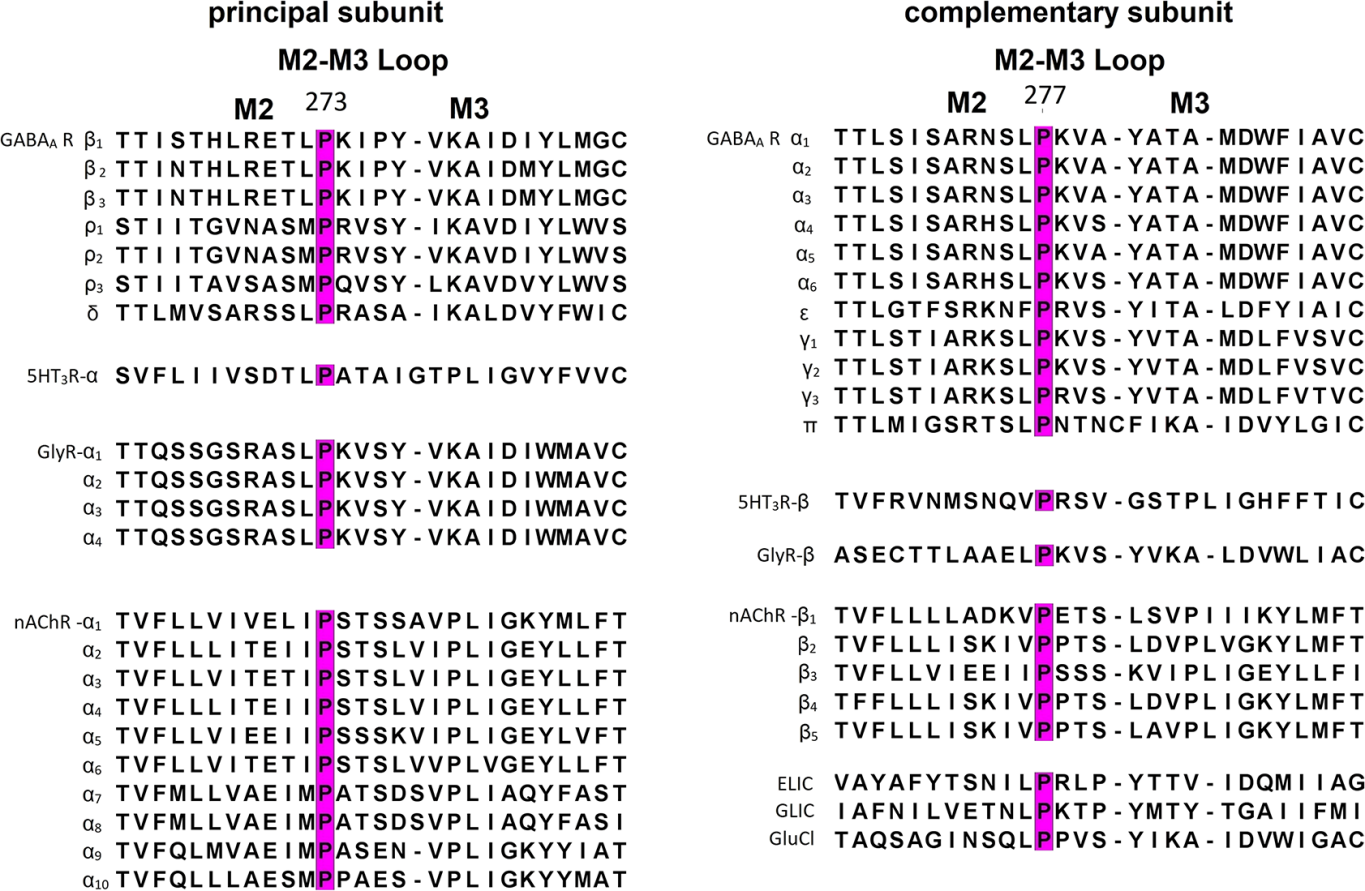
Alignment of the Cys-loop receptors’ M2-M3 loop region sequence. Note that the proline residue located at the M2-M3 loop is highly conserved in both principal and complementary subunits across different types of Cys-loop receptors, including those in *Eukaryota* and *Prokaryota* species.

**Figure 2:**
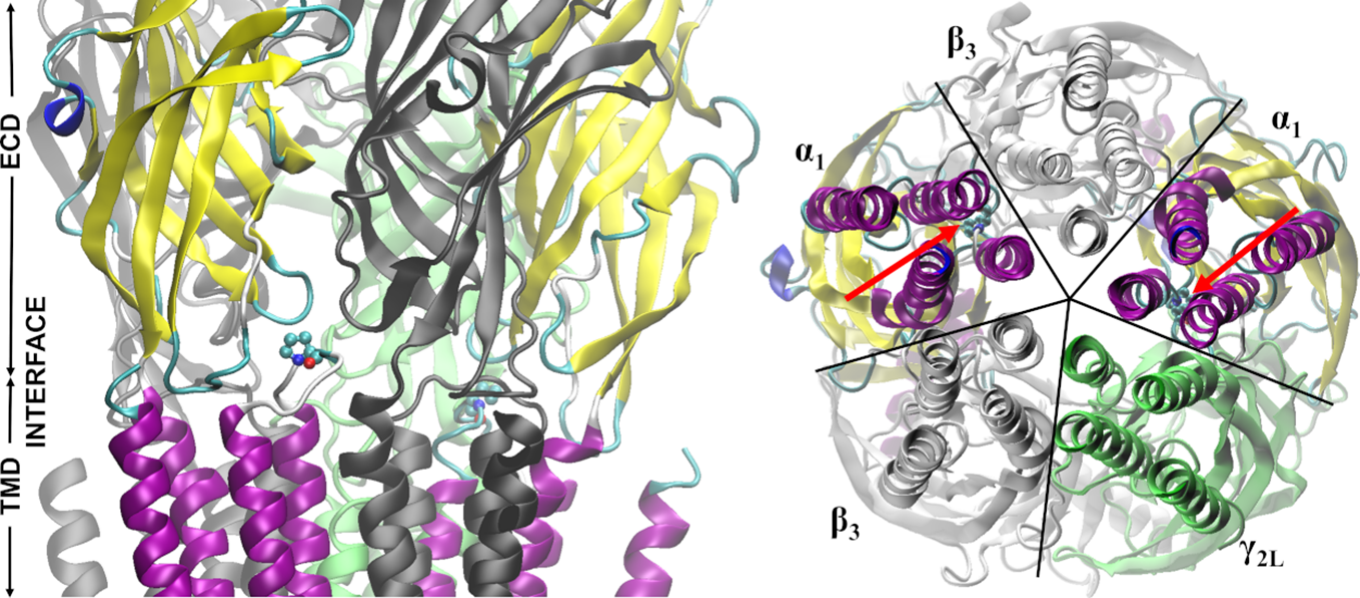
Location of P277 in GABA_A_R. The proline 277 residue (pointed with a red arrow) is localized on the α subunit in the interface region of GABA_A_R, where many structures from the ECD and TMD are interacting. α subunits are highlighted with colorization of its secondary structures: α-helices were marked with purple tones, β-sheets with yellow tones, loops with cyan/white tones, β subunits with gray tones, and γ subunit with green tones.

Interestingly, the M2-M3 loop/linker of the GABA_A_R interface was found to be important for not only the receptor activation^4^ but also in the context of benzodiazepine/volatile anesthetic action.^38,39^ For the M2-M3 loop, the most conserved amino acids through all types of Cys-loop receptors (both in principal and complementary subunits) are prolines 273 and 277 (numeration based on the cDNA coding α_1_ subunit for *Rattus norvegicus*, 278 for humans, Figure 1)*.* This local interaction mediated by proline is related to the physicochemical properties of the pyrrolidine ring and its thermodynamic equilibrium between *cis*- and *trans*-isomers.^40−42^ The presence of well-conserved prolines in the M2-M3 linker is particularly intriguing, especially considering their stiffening impact on protein local flexibility^43,44^ and their strategic location, potentially influencing interactions with neighboring structures within the interface region (Figure 2). Moreover, single proline residues found in the amino acid sequence defining the loop structure (around 9–10 residues) have a stabilizing effect on its formation kinetics and its physicochemical properties.^45^ Considering these premises, it can be expected that proline within the M2-M3 linker plays a key role in determining the flexibility of this loop.^35^ The importance of P277 residues for GABA_A_Rs was hinted by Bera and co-workers^34^ who found that substitution of P277 with cysteine caused a relatively strong rightward shift in dose–response together with amplitude reduction. A rightward shift in the dose–response was also reported when substituting P277 with alanine,^38^ but the change in EC_50_ for GABA was much smaller than that reported by Bera and co-workers for the cysteine mutation. It remains unclear to what extent the mutation of the P277 residue affects the receptor gating. Indeed, as pointed out by Colquhoun,^46^ shifts in the dose–responses could result from alterations in both agonist affinity and gating. It is also worth noting that, as pointed out in the study of Woll et al.,^39^ the P277 residue is likely to interact with volatile anesthetic, suggesting that this residue can be crucial in the GABA_A_R modulation by these compounds. Overall, the present evidence clearly indicates that proline 277 within the M2-M3 loop is strongly involved in the receptor activation and also in the receptor modulation by clinically relevant compounds. However, which specific gating transitions are affected by this residue and what are the underlying structural determinants of its role remain basically unknown. To address this issue, we have considered α_1_β_2_γ_2L_ receptors with α_1_P277 substitutions. In an attempt to shed light on the mechanistic picture of how this residue is involved in shaping the receptor gating properties, we have used substituting amino acids showing a wide spectrum of physicochemical properties: small alanine, lysine, and glutamic acid that carry electrostatic charge on their side chains and, last, histidine with its characteristic imidazole ring. Our electrophysiological investigations together with extensive modeling revealed that P277 at the M2-M3 loop is strongly involved in controlling practically all transitions of the receptor gating.

## Results

### Impact of α_1_P277 Mutation on Macroscopic Currents

**Figure 3:**
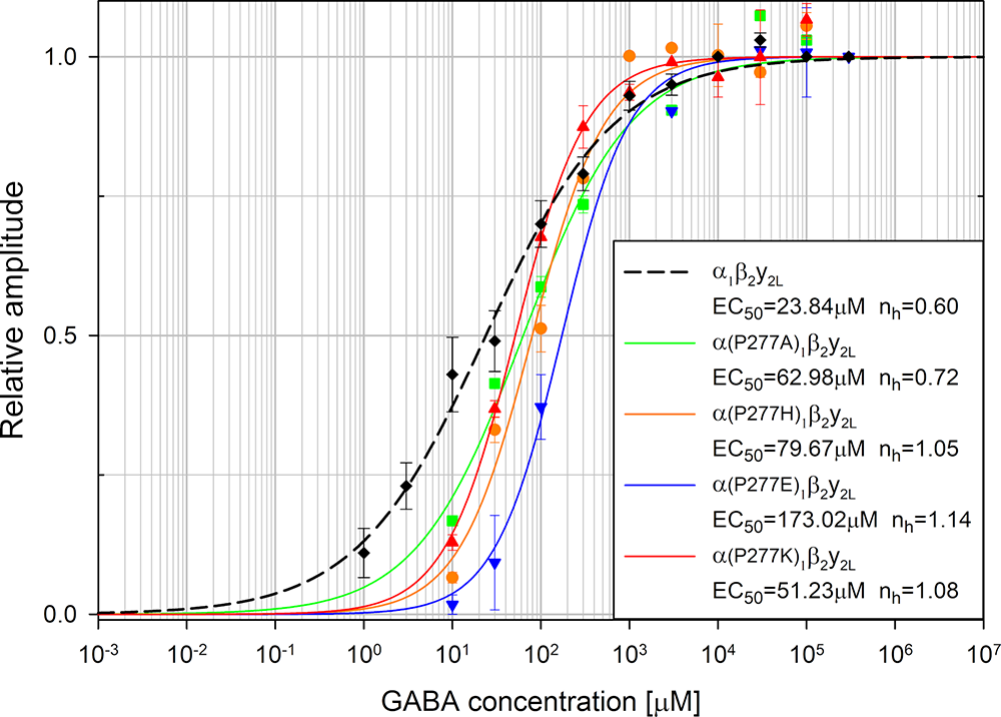
α_1_P277 substitutions cause slight rightward shifts in dose–responses with respect to WT. Comparison of dose–response relationships, normalized to amplitude for sat. [GABA] for P277 mutations (P277A, green; P277E, blue; P277H, orange; P277K, red) and to dose–response assessed for WT (black symbols and black dashed curve).

Effects of P277 mutations were first investigated by constructing the dose–response relationships. For all mutants except for P277E, the macroscopic currents were measured in the excised patch configuration that assures high temporal resolution. In the case of P277E, the dose–response was constructed from currents recorded from lifted cells (whole-cell) because of low expression. For all of the considered mutants, the 10 mM concentration of GABA was sufficient to reach saturation (Figure 3).

As shown in Figure 3, mutations of the P277 residue had a relatively weak effect on the dose–response, resulting in a small rightward shift in comparison to the WT receptors (the dashed line in Figure 3 represents Hill’s curve plot for WT receptors obtained previously by our group^47^ in the same experimental conditions). The smallest change in EC_50_ value was observed for P277K (EC_50_ = 51.23 μM) and the largest one was observed for P277E (EC_50_ = 173.02 μM), while P277A (EC_50_ = 62.98 μM) and P277H (EC_50_ = 79.67 μM) were closer to P277K than to P277E. Overall, this analysis suggests that mutations at the P277 residue have a minor effect on agonist binding. It remains, however, to be elucidated to what extent this residue is involved in regulation of the receptor gating. To this end, we have analyzed the kinetics of current responses elicited by saturating [GABA] (10 mM for all mutants).

**Figure 4:**
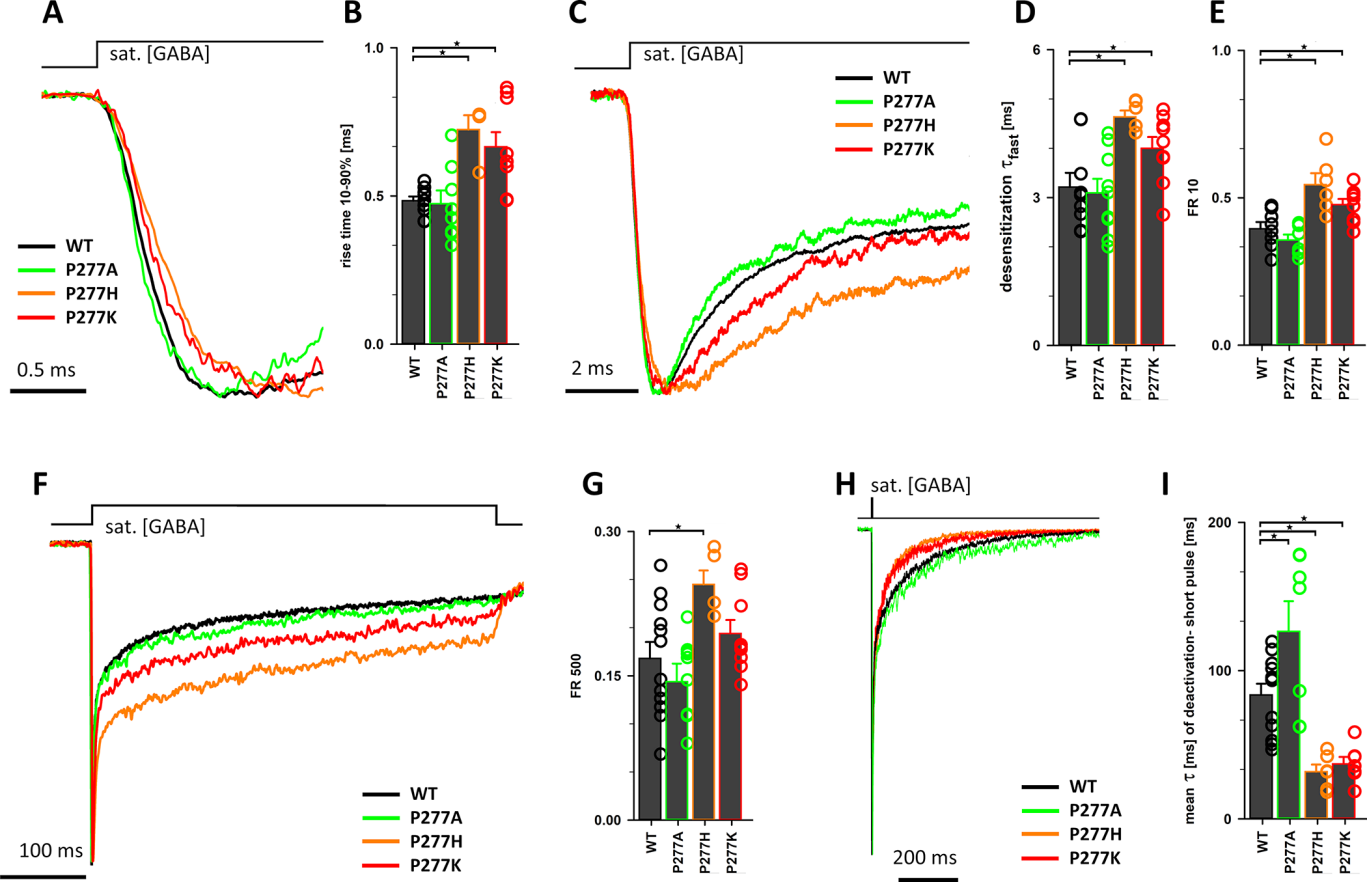
P277 mutations alter macroscopic currents, evoked by sat. [GABA], indicating alterations in receptor gating. Traces for WT (black), P277A (green), P277H (orange), and P277H (red) were normalized to better visualize the differences in the time course of currents. (A) Examples of the current rising phases and the statistics for RT (10–90% of onset time) are shown in panel (B). In panel (C), traces are shown in the timescale revealing rapid macroscopic desensitization and panels (D) and (E) present statistics for the rapid desensitization component assessed as the time constant (τ_fast_) or the FR10 parameter. In panel (F), traces are presented in the large timescale showing biphasic macroscopic desensitization, and statistics for the FR500 parameter is shown in panel (G). (H) Current traces recorded in response to application of 2 ms pulse of sat. [GABA] (protocol to reveal deactivation) and (I) statistics for the weighted deactivation time constant (mean τ). Statistically significant differences between each case are presented with the inset above bars with asterisks (“*”). Insets above current traces indicate the timing of agonist applications.

As already mentioned, in the case of the P277E mutant, recordings were made from the lifted cells because of low expression, these recordings were characterized by markedly lower time resolution compared to those in excised patches, and for this reason, the macroscopic kinetic analysis was not performed for this mutant. As shown in Figure 4A,B, the current onset (RT, 10–90%) was significantly prolonged for both P277H and P277K (respectively: 0.72 ± 0.04 ms, *n* = 4; 0.67 ± 0.06 ms, *n* = 6, *p* < 0.05 in each case) when compared to WT (0.48 ± 0.01 ms, *n* = 10). However, in the case of the P277A mutant, the rising phase of current responses was undistinguishable from that measured for the WT receptors (Figure 4A,B). A similar trend was observed for parameters describing rapid desensitization: P277H and P277K mutations resulted in a slow-down of the τ_fast_ time constant (Figure 4C,D; P277H: 4.64 ± 0.13 ms, *n* = 6; P277K: 4.15 ± 0.23 ms, *n* = 6; *p* < 0.05 for both) compared to WT (3.21 ± 0.29 ms, *n* = 9) and in increased FR10 (P277H; 0.55 ± 0.04, *n* = 6; P277K: 0.48 ± 0.03, *n* = 6; WT: 0.39 ± 0.02, *n* = 10; *p* < 0.05 for both mutations; Figure 4E). To assess the extent of desensitization induced by long application, the FR500 parameter was used and we observed that P277 mutation tended to increase this parameter (Figure 4F), but a statistically significant difference was observed only for P277H (P277H: 0.25 ± 0.01, *n* = 5; *p* < 0.05; P277K: 0.19 ± 0.02, *n* = 6; *p* = 0.48; WT: 0.17 ± 0.02, *n* = 12; Figure 4G). We have also analyzed the deactivation time course (current decay after 2 ms pulse of saturating [GABA], Figure 4H,I). Both P277H and P277K mutations caused a strong and similar acceleration of the deactivation time constant (P277H: 31.92 ± 4.74 ms, *n* = 6; P277K: 37.14 ± 6.56 ms, *n* = 5) when compared to WT (83.66 ± 7.50 ms, *n* = 12, *p* < 0.05 for comparison with both mutants). In contrast, the deactivation time course for P277A mutants was slightly but significantly prolonged with respect to the WT receptors (P277A: 126.47 ± 20.34 ms, *n* = 7, *p* < 0.05; Figure 4H,I).

As explained below, model simulations for macroscopic currents required a strict confrontation with the single-channel data and, for this reason, will be presented after the sections dedicated to single-channel recordings.

### Global Effect of P277 Mutation on GABA_A_R Single-Channel Gating Kinetics

**Figure 5:**
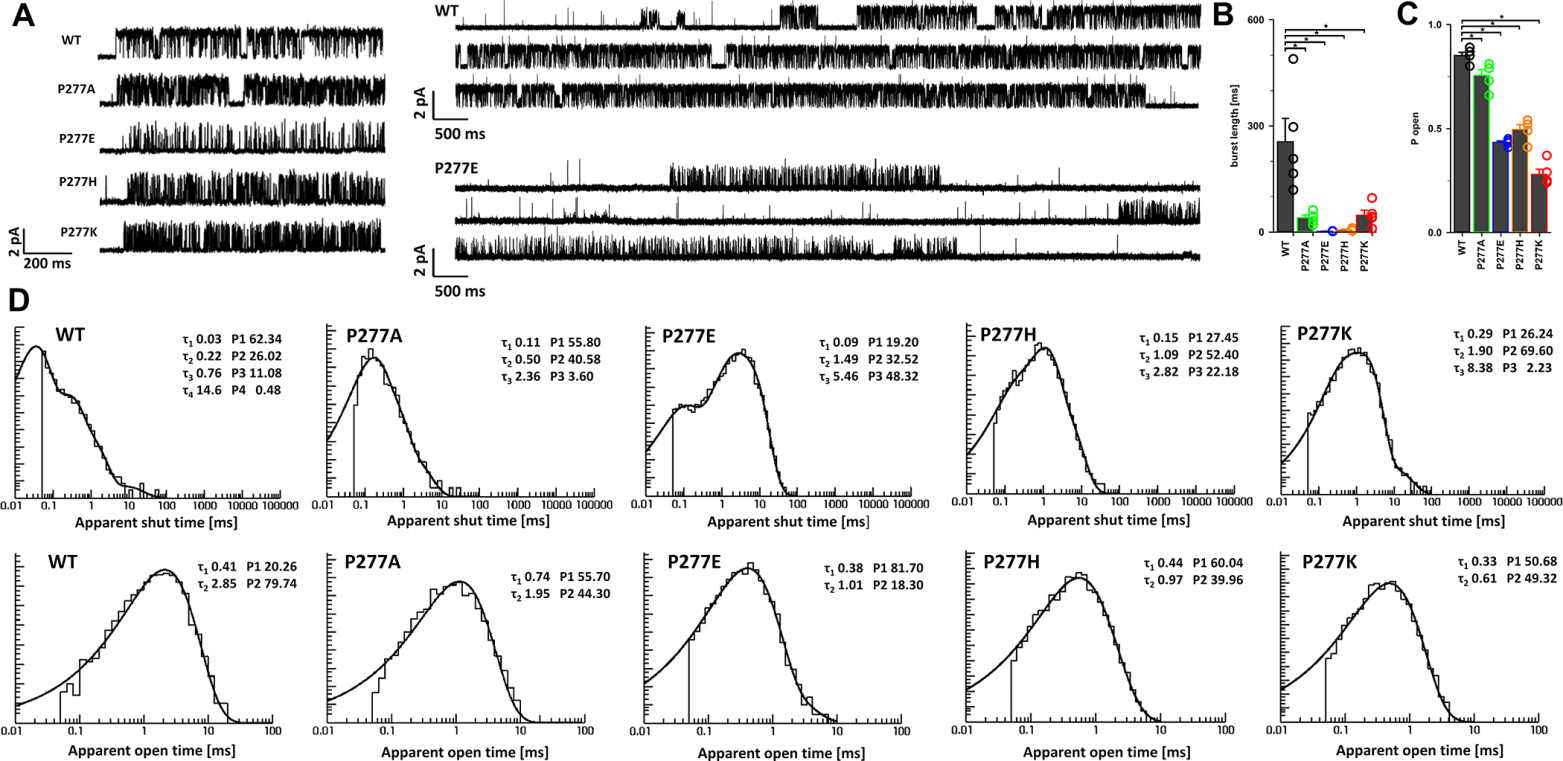
Single-channel event experimental distribution is affected by P277 mutations. (A) Examples of typical activity for WT and P277A, P277E, P277H, and P277K and examples of recordings in a larger scale for WT and P277E. Mean burst lengths (*B*) and *P*_open_ values for bursts (C) for P277 mutants were significantly shorter than for those for WT. (D) Typical examples of experimental shut and open time distributions for WT and P277 mutants.

To further investigate the impact of P277 substitution in the M2-M3 loop, single-channel recordings were carried out in conditions of saturating [GABA] in the cell-attached configuration. For each substitution (P277A, P277E, P277H, and P277K), a clear cluster activity (Figure 5A) was observed with different modes of activity, similar to what was reported in earlier works.^8,48,49^ Dominant activity mode as described in Materials and Methods was used for analysis, and the analysis presented below concerns the dominant mode. For each single-channel recording, after calculating the signal-to-noise ratio (see Materials and Methods), clusters representing dominant activity mode were individually analyzed with the use of different resolutions ranging from 50 to 90 μs. The final resultion used in the analysis was choosen for each recording based on the exponential function aligment (describing shut/open times) to the histogram representing event (shut/open time) distribution. In each case, the resolution of 50 μs allowed for the best description of event time distribution by exponential functions; thus, experimental resolution was set as 50 μs in the single-channel analysis of the dominant modes of WT and P277 mutants. In each of the considered mutations, a statistically significant shortening of burst length (Figure 5B) and *P*_open_ within the bursts (Figure 5C) was observed compared to WT: for P277A (40.61 ± 7.19 ms and 0.75 ± 0.03, respectively, *n* = 5; *p* < 0.05 for burst length and *P*_open_ compared to WT), P277E (2.31 ± 0.17 ms, 0.43 ± 0.01, *n* = 5; *p* < 0.05), P277H (6.59 ± 1.64 ms, 0.50 ± 0.02, *n* = 5; *p* < 0.05), P277K (48.03 ± 13.91 ms, 0.28 ± 0.02, *n* = 5; *p* < 0.05), and WT (255.42 ± 65.37 ms, 0.85 ± 0.02, *n* = 5).

For P277 mutants and WT receptors, open times were fairly characterized by two components. However, in the case of mutations, we have found that three shut time components were sufficient for shut time description vs four components in WT. Notably, all the time constants for shut times in mutants are roughly 3–10-fold slower than the respective ones for WT (Figure 5D and Table 1), which is consistent with observed *P*_open_ reduction. We thus performed comparisons of time constants for mutants with the respective three shortest components for WT. Considering the above-mentioned trend, it is likely that the slowest shut time constant in mutants increased so much that it went undetected in the steady-state recordings as it could fall into the end-cluster shut time range. This possibility would be consistent with a strong reduction in cluster durations in mutants. On the other hand, it is also possible that the slowest shut time component could simply disappear because of the reshaping of the receptor gating scheme upon mutation. As shown in Table 1, P277 mutations strongly affected both the time constants and percentages of the shut time components with an overall increase in mean shut time. In particular, we observed a prominent increase and lowering of the percentage for second and third components for P277A and P277K, respectively. On the other hand, P277H and P277E mutations were characterized by a large increase in both second and third components. Moreover, as shown in Table 2, mutations resulted in a strong shortening of the open times, with most substantial changes in the second component and in percentages of both of them. In particular, in contrast to WT, the short opening component became predominant in all mutants.

**Table 1:** Experimental and Simulated (0 μs res, in Square Brackets) Values of Distribution Parameters for Shut Times for P277 Mutants and WT^a^

	*P*_1_	τ_1_ [ms]	*P*_2_	τ_2_ [ms]	*P*_3_	τ_3_ [ms]	*P*_4_	τ_4_ [ms]	Τ_shut_ [ms]
WT	62.34 ± 0.84	0.030 ± 0.001	26.02 ± 1.82	0.22 ± 0.12	11.08 ± 2.04	0.76 ± 0.04	0.48 ± 0.15	4.62 ± 3.15	0.23 ± 0.21
0 μs	[58.40 ± 2.21]	[0.033 ± 0.001]	[30.30 ± 1.8]	[0.24 ± 2.62]	[10.7 ± 2.54]	[0.97 ± 0.14]	[0.60 ± 0.002]	[14.32 ± 3.16]	[0.26 ± 0.03]
P277A	55.80 ± 4.02*	0.11 ± 0.01*	40.58 ± 4.12*	0.50 ± 0.05*	3.60 ± 0.76*	2.36 ± 0.38*			0.34 ± 0.04*
0 μs	[54.90 ± 5.66]	[0.11 ± 0.02]*	[42.70 ± 5.18]	[0.53 ± 0.06]*	[2.40 ± 0.51]*	[3.32 ± 0.56]*			[0.35 ± 0.04]
P277E	19.20 ± 1.07*	0.09 ± 0.02*	32.52 ± 1.98*	1.49 ± 0.07*	48.32 ± 2.52*	5.46 ± 0.41*			3.12 ± 0.17*
0 μs	[19.90 ± 0.009]*	[0.1 ± 0.02]*	[45.90 ± 9.36]	[1.97 ± 0.43]*	[34.20 ± 9.51]*	[7.53 ± 1.26]*			[3.20 ± 0.17]*
P277H	27.45 ± 0.56*	0.15 ± 0.01*	52.40 ± 4.83*	1.09 ± 0.07*	22.18 ± 3.97*	2.82 ± 0.11*			1.23 ± 0.09*
0 μs	[26.60 ± 2.50]*	[0.16 ± 0.01]*	[62.80 ± 0.03]*	[1.32 ± 0.21]*	[10.60 ± 4.21]*	[4.11 ± 0.59]*			[1.24 ± 0.09]*
P277K	26.24 ± 1.51*	0.29 ± 0.04*	69.60 ± 1.97*	1.90 ± 0.13*	2.23 ± 0.60*	8.38 ± 1.40*			1.71 ± 0.10*
0 μs	[26.60 ± 1.59]*	[0.3 ± 0.04]*	[69.00 ± 2.01]*	[1.90 ± 0.13]*	[4.40 ± 2.22]	[12.97 ± 4.75]*			[1.7 ± 0.1]*

a Each mean value was obtained from five cells. Statistical significance differences with respect to WT were marked in boldface and with an asterisk (“*”) on the right side of the value.

**Table 2:** Experimental and Simulated (0 μs res, in Square Brackets) Values of Distribution Parameters for Open Times for P277 Mutants and WT^a^

	*P*_1_	τ_1_ [ms]	*P*_2_	τ_2_ [ms]	Τ_open_ [ms]
WT	20.26 ± 3.86	0.41 ± 0.09	79.74 ± 3.86	2.85 ± 0.15	2.47 ± 0.12
0 μs	[23.10 ± 7.15]	[0.72 ± 0.26]	76.90 ± 7.15	2.87 ± 0.13	[2.48 ± 0.13]
P277A	55.7 ± 14.64*	0.74 ± 0.2	44.3 ± 14.64*	1.95 ± 0.24*	1.29 ± 0.11*
0 μs	[68.00 ± 8.39]*	[0.94 ± 0.14]	[32.00 ± 0.08]*	[2.12 ± 0.24]*	[1.29 ± 0.11*]
P277E	81.7 ± 5.5*	0.38 ± 0.03	18.3 ± 5.5*	1.01 ± 0.08*	0.48 ± 0.03*
0 μs	[82.70 ± 5.50]*	[0.42 ± 0.006]	[17.20 ± 0.06]*	[1.1 ± 0.15]*	[0.51 ± 0.03*]
P277H	60.04 ± 16.04*	0.44 ± 0.04	39.96 ± 16.04*	0.97 ± 0.14*	0.60 ± 0.02*
0 μs	[78.70 ± 8.11]*	[0.51 ± 0.04]	[21.30 ± 0.08]*	[1.07 ± 0.13]*	[0.60 ± 0.02*]
P277K	50.68 ± 5.8*	0.33 ± 0.04	49.32 ± 5.8*	0.61 ± 0.02*	0.45 ± 0.01*
0 μs	[56.90 ± 10.40]*	[0.33 ± 0.05]	[43.10 ± 0.1]*	[0.65 ± 0.045]*	[0.48 ± 0.01*]

a Each mean value was obtained from five cells. Statistical significance differences with respect to WT were marked in boldface and with an asterisk (“*”) on the right side of the value.

### Single-Channel Modeling Reveals that P277 Is Crucially Involved in GABA_A_R Gating

**Figure 6:**
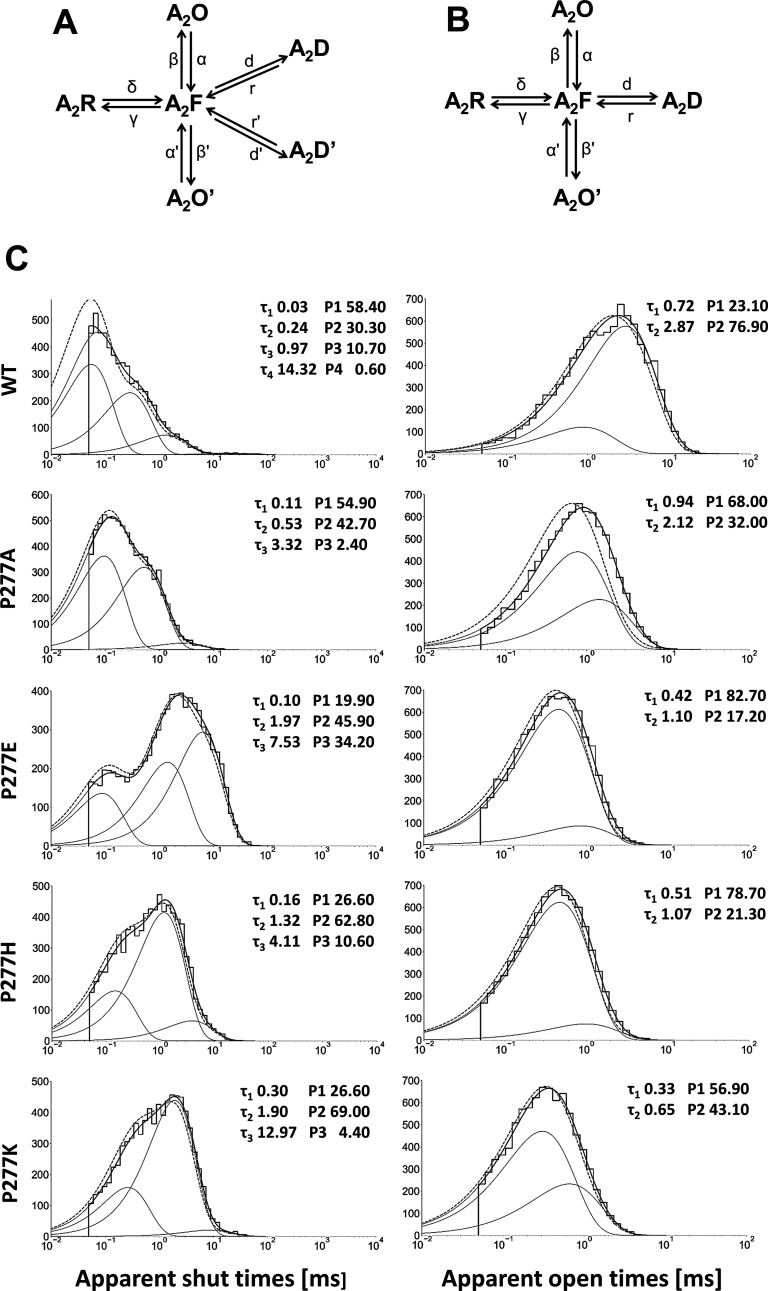
Model simulation for single-channel measurements. Models for simulation of single-channel activity were based on the flipped Jones–Westbrook model with the omitted binding step to account for sat. [GABA]. The activity of WT was simulated with model (A) and P277A mutants with model (B). Panel (C) shows simulated (solid black line) and idealized (gray dashed line) event distribution for WT and P277 mutations together with components (gray solid line).

To investigate the impact of P277 substitution on GABA_A_ single-channel kinetic properties, we used kinetic models with the ommited binding step (Figure 6A,B) as, in these experiments, GABA was continuously present at a saturating concentration (10 mM, see also refs (8) and (49)). Considering that for WT receptors, consistently four shut time components were found, and the model with four shut states was used (A_2_R, A_2_F, and two desensitized states A_2_D and A_2_D′, Figure 6A). However, for P277 mutants (three shut time components), the model containing only one desensitized state was used instead (Figure 6B). Kinetic rates obtained from modeling (Table 3) allowed for good reproduction of closure and opening distributions (see exemplary distributions in Figure 6C) and of idealized opening and shut times with respective percentages were comparable to experimental ones (Tables 1 and 2, rows starting with 0 μs). Intriguingly, nearly all kinetic rates were affected by considered mutations (Table 3). However, the biggest changes were observed for β, β′, and δ rates. The only rate constants not significantly affected by mutations were as follows: α for P277E and P277K, γ for P277E, and the desensitization rate *d* for P277E. Notably, α′ values were increased roughly 2-fold, while the γ rate was reduced from two to four times. Taken altogether, the results of this modeling show that mutation of the P277 residue results in an overall change in the receptor gating with major effects on flipping and open/shut transitions. In addition, the rate constants describing desensitization (*d* and *r*) determined from single-channel data were different from those obtained in macroscopic simulations (graph in Figure 5). As discussed in our previous reports,^8,23^ this discrepancy results primarily from distinct recording conditions (nonstationary in the macroscopic channel and steady state in the single channel).

**Table 3:** Kinetic Rate Constants Describing Single-Channel (s-ch) Cluster Activity for WT and P277 Mutants

s-ch kinetic rate constants [ms^–1^]	WT	P277A	P277E	P277H	P277K
δ	5.82 ± 0.53	3.15 ± 0.44*	1.77 ± 0.22*	2.19 ± 0.34*	1.4 ± 0.13*
γ	7.12 ± 0.29	2.63 ± 0.53*	6.76 ± 1.17	2.74 ± 0.36*	1.43 ± 0.33*
α	2.77 ± 0.58	0.7 ± 0.15*	1.36 ± 0.37	1.02 ± 0.14*	1.62 ± 0.11
β	13.82 ± 1.77	2.5 ± 1.32*	0.76 ± 0.25*	0.39 ± 0.14*	0.63 ± 0.19*
α′	0.64 ± 0.05	1.34 ± 0.28*	2.65 ± 0.06*	2.21 ± 0.14*	3.52 ± 0.59*
β′	14.08 ± 0.91	4.45 ± 1.27*	2.26 ± 0.63*	2.13 ± 0.22*	0.9 ± 0.1*
*d*	1.81 ± 0.47	0.13 ± 0.03*	1.05 ± 0.42	0.14 ± 0.06*	0.02 ± 0.006*
*r*	1.22 ± 0.18	0.34 ± 0.05*	0.23 ± 0.06*	0.29 ± 0.05*	0.12 ± 0.04*
*d*′	0.11 ± 0.03				
*r*′	0.09 ± 0.03				

Rate constants were determined for a saturating concentration of GABA with model A for WT and model B for P277 mutants (Figure 6). Significant changes in rate constants relative to WT are marked in boldface and with an asterisk (*). For each considered case (WT and mutation), the data were obtained from five patches.

### Macroscopic Current Simulations Largely Confirm the Impact of P277 Mutations on Receptor Gating

**Figure 7:**
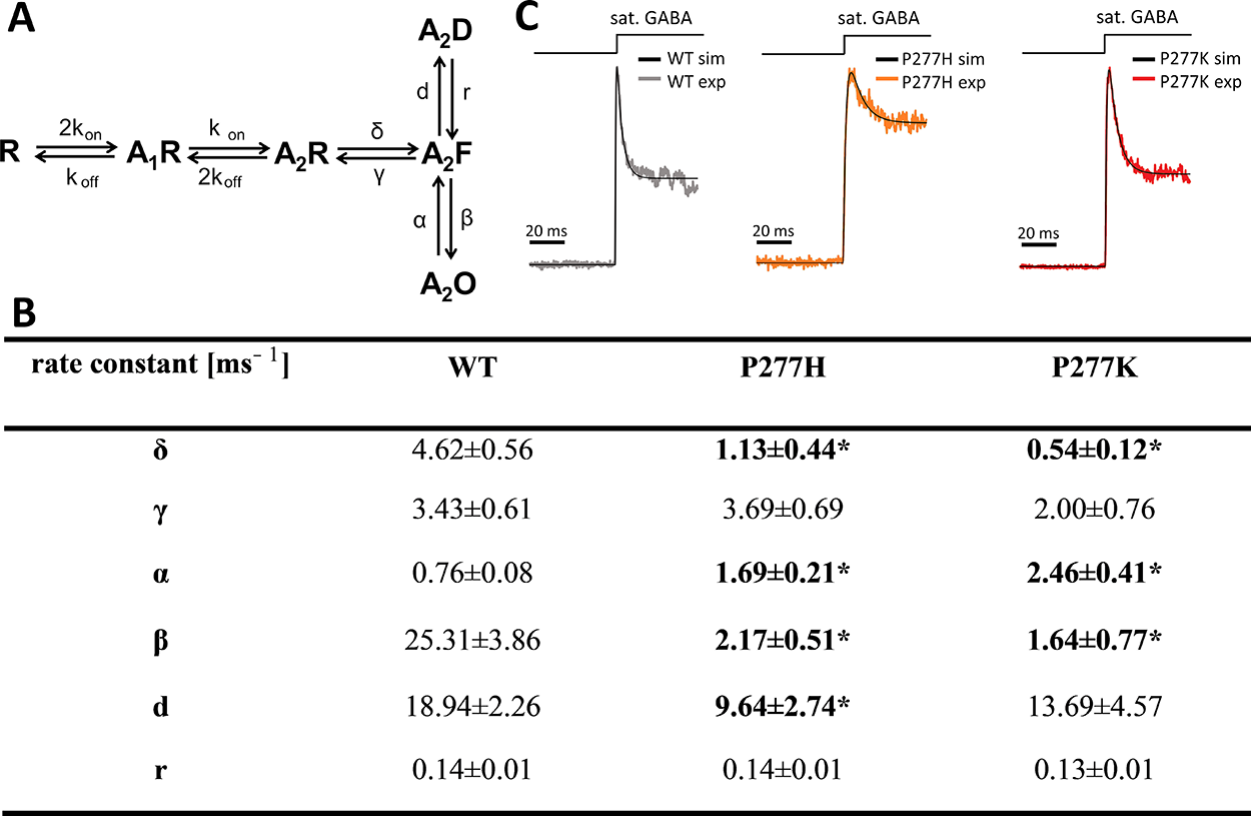
Modeling of macroscopic current responses. Simulation for evoked currents was performed with the flipped Jones–Westbrook model (A). Since all experiments were performed under sat. [GABA] conditions, values of the rate constants describing the binding steps were taken from ref (50) and fixed in simulations. Additionally, since dose–responses for WT and mutants differed slightly (Figure 3), the same *k*_on_ and *k*_off_ values were used for all of these receptors. Panel (B) shows rate constants for WT, P277H, and P277K. In each case, mean values of the rate constants were obtained from fitting at least four current responses. Significant changes in values of the rate constants were indicated in boldface and with an asterisk (“*”). Panel (C) presents experimental current responses for WT, P277H, and P277K together with fitted traces using model (A). Insets above traces represent sat. [GABA] administration.

To further explore the impact of the α_1_P277 residue mutation on the receptor function, we performed additional model simulations for macroscopic currents. For this purpose, the flipped Jones–Westbrook model (Figure 7A), previously proposed by our group,^50^ was used. Experimental current responses were fitted by optimizing the rate constants in the model using ChannelLab software (Figure 6B). Since the considered model (Figure 7A) has only one desensitized state, we limited fitting to the time window in which the fast component of macroscopic desensitization was predominant (approx. 30 ms, Figure 7B). Each cell for which the current response to long sat. [GABA] application was measured contributed to statistics with one complete set of the rate constants. Upon modeling the experimental current traces for mutants, we have encountered a difficulty that, although fitting with the considered model converged, the values of the optimized rate constants strongly depended on the initial values. For instance, in the case of the α_1_P277H mutant, for some initial values, the observed current phenotype could be well reproduced by a decrease in either α or *d* rate constant only. However, a decrease in α rate constant (exit from the open state) would predict that the mean open time of the α_1_P277H mutant would increase, contrary to the single-channel data (Table 2). Thus, we decided to choose for the ChannelLab fitting of the starting values of the flipping/unflipping and opening/closing rates (δ, γ, α, and β) determined from the single-channel analysis (Table 3), while initial values for *d* and *r* were taken from the macroscopic analysis of WT receptors. Considering that models employed in single-channel analysis had two open states, for macroscopic modeling (one open state), the average values of α and β rate constants from single-channel modeling were taken as initial values. As explained in detail in our previous reports,^9,24^ estimations for *d* and *r* rate constants from stationary single-channel analysis yield markedly different values of these rate constants because of distinct experimental conditions. The resulting rate constants for the α_1_P277H mutant, estimated with ChannelLab for these initial values, are presented in Figure 7C.

The same approach was applied to the α_1_P277K mutant, but data for α_1_P277A substitution were not modeled because of high similarity to responses mediated by WT receptors. Overall, our macroscopic data and modeling confirm that both examined mutations of the α_1_P277 residue (α_1_P277H and α_1_P277K) affect the transition into the flipped state (decrease in δ rate) and opening/closing rates (decrease in β rate and increase in α rate), and additionally, in the case of the α_1_P277H mutant, also the desensitization rate was affected (decrease in *d*).

## Discussion

### P277 Residue Is Critically Involved in GABA_A_R Gating but Not Binding

The major conclusion of the present work is that mutations of the α_1_P277 residue strongly affect nearly all transitions of the receptor gating, having a relatively minor effect on the binding reaction. Our analyses of single-channel activity and macroscopic current responses provided consistent evidence that preopening (flipping) and open/close transitions are particularly strongly affected by this mutation (Tables 1 and 2). Surprisingly, in the steady-state single-channel recordings, all the rate constants for the considered substitutions (except only for *d* for α_1_P277E) describing desensitization (*d* and *r*) were significantly affected by the mutations (Table 3), whereas the relative effect on *d* and *r* rate constants appeared more moderate in the case of macroscopic recordings (only *d* for α_1_P277H was significantly altered, Figure 7B). This is intriguing because, as discussed in our previous reports,^8,9,23,24,51^ macroscopic recordings of responses to rapid applications of the saturating agonist offer optimal conditions to reveal the desensitization onset, whereas steady-state measurements are carried out in conditions in which the majority of receptors are desensitized. For this reason, stationary single-channel recordings typically do not reveal rapid desensitization rate constants, which also explains differences in their estimation in macroscopic and single-channel recordings (see Table 3 and Figure 7B). A more pronounced difference in desensitization rate constants in single-channel recordings compared to macroscopic measurements in the present study reflects most likely a higher accuracy of the former ones in this set of experiments.

Our conclusion regarding the limited impact of α_1_P277 substitutions on the agonist binding is based on the small rightward shift of the dose–response relationships (Figure 3). Notably, weak effects on the dose–response were observed for substituting residues strongly differing in their physicochemical properties: small and neutral alanine and amino acids charged with negative or positive charge (glutamate or lysine). This observation clearly indicates that α_1_P277 has a weak impact on agonist binding reaction being primarily involved in regulating the receptor gating. A minor involvement of α_1_P277 on the agonist binding is not surprising considering a large distance between this residue and the binding cassette. Similarly, our previous studies on substitutions at residues distant from the orthosteric binding site, in the ECD-TMD interface^8^ or in the transmembrane domain,^9^ also reported the weak impact of these mutations on the agonist binding. On the other hand, it is worth noting that dose–response relationships for point mutations of the H56 residue (numbering derived for the cDNA coding α_1_ subunit for humans), which is in close proximity to P277, were characterized by shifts correlated with the side-chain charge: leftward for lysine and rightward for glutamate.^52^ Thus, agonist binding could show some sensitivity to molecular interactions occurring between residues in the vicinity of the interface, but the overall effect is typically limited.

The impact of α_1_P277 mutation on the receptor gating is supported in our macroscopic recordings by the observations that the considered substitutions altered practically all characteristics of these currents including their onset, macroscopic desensitization, and deactivation (Figure 4). Considering that the agonist concentration was saturating, these observations are attributed to changes in the kinetics of conformational transitions between fully bound states (gating). Our macroscopic modeling revealed that nearly all the rate constants are altered by the considered substitutions (P277H and P277K; Figure 7B), which appears consistent with observed alterations of all parameters describing the time course of macroscopic current responses (Figure 4). The notion that mutation of the α_1_P277 residue causes a global change in the receptor gating is further reinforced by our single-channel analysis. Indeed, just the appearance of the single-channel traces elicited by saturating [GABA] (Figure 5A) together with dramatic changes in burst durations and open probability (Figure 5B,C) reveals obvious changes in the receptors’ gating caused by the considered mutations. Furthermore, as shown in Tables 1 and 2, the vast majority of parameters describing the features of shut and open time distributions are significantly affected. It is thus not surprising that single-channel modeling revealed profound changes in gating properties manifested by alterations of nearly all the rate constants included in the models (Table 3). This observation suggests that the α_1_P277 residue is involved in transduction of the molecular signal related not only to any specific transition but rather to some complex information transfer relevant to all types of conformational changes. It is worth emphasizing that a similar *modus operandi* has been observed also for a number of other residues throughout the structure of the GABA_A_R macromolecule. Indeed, mutations of, e.g., binding site α_1_F64, β2F200,^23,53^ peripheral α_1_F14, β2F31,^51^ interface-located α_1_H55,^8^ and transmembrane M2 and M3 helices β2G254V, α_1_G258V, α_1_L300V, and β2L296V^9^ affected most of the gating transitions. The most remarkable in this context is the observation that microscopic desensitization of the GABA_A_ receptor is highly sensitive to mutations of residues in most of localizations studied by our group thus far.^8,9,23−25,50,51^ This feature of desensitization led us to propose the concept of a “diffuse desensitization gate”^8,9^ as opposed to the desensitization gate largely restricted to the receptor’s transmembrane domains.^54^ In general, the emerging picture is that structural determinants of various conformational transitions are not compartmentalized, but rather specific elements of the protein structure are being shared upon distinct phases of the receptor activation. This concept of widespread structural gating mechanisms appears to hold also with respect to the α_1_P277 residue as it turns out to be important in all conformational transitions included in the considered gating scheme. Thus, the results of the present study reinforce the view that the ECD-TMD interface plays a role of a key “gating transducer” with the α_1_P277 residue being its important element. The impact of the interface region in GABA_A_R gating has been proposed also in previous studies;^32,33^ however, we have extended this information to specific gating transitions such as preactivation, opening/closing, and desensitization. An important role of the interface has been also proposed for other Cys-loop receptors such as nAChR^55,56^ or GlyR.^57^

An important and still unresolved issue is the molecular mechanisms underlying the role described here of the α_1_P277 residue in GABA_A_R gating. Our results based on α_1_P277 substitutions with charged amino acids with opposite charges show that, in the case of this residue, the receptor gating is weakly sensitive to the side-chain electrostatics. Interestingly, in the case of a nearby α_1_H55 residue, we observed a clear dependence of macroscopic currents’ features (onset and macroscopic desensitization) on the side-chain charge of amino acid substituting the histidine.^8^ Thus, even for residues in close proximity within the ECD-TMD interface, different molecular scanarios determine their impact on the receptor gating. In general, it seems that the extent of changes in GABA_A_R gating (or other Cys-loop reeceptors) caused by substitutions of residues in the interface region can be either due to altered steric interactions between neighboring structures^29,30^ or changes in the electrostatic properties.^31,32,58^ Our data would thus indicate that the role of P277 in shaping the receptor gating may be limited to steric interactions and its impact on the protein backbone.

**Figure 8:**
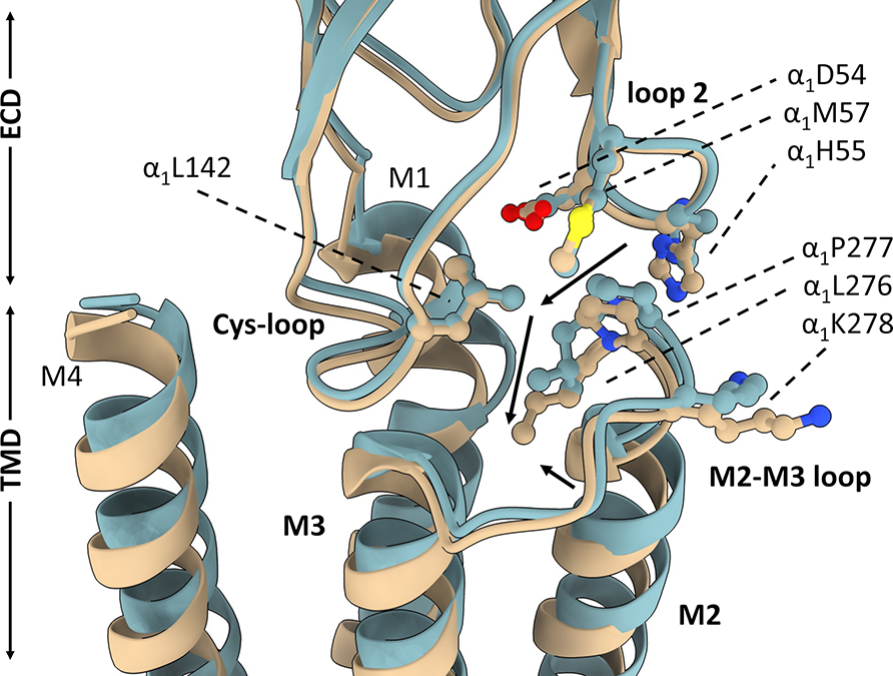
Structure of the ECD/TMD interface. Superimposed α_1_ subunits of GABA_A_R shown in brown (structure in the shut state with bicuculine bound; PDB code: 6X3S) and blue colors (structure in the desensitized state with GABA bound; PDB code: 6X3Z). P277 and neighboring residues shown in ball-and-stick representation. Arrows indicate the movement of the residues between desensitized and shut states.

Considering the above-described functional impact of α_1_P277 mutations, we made an attempt to indicate possible local molecular interactions of this residue and their likely consequences in the context of the present findings. As shown in Figure 8, the α_1_P277 residue is located at the M2-M3 loop being surrounded by α_1_L276 (which points its functional group toward the subunit’s transmebrane helix bundle) and α_1_K278 (which is oriented toward the neighboring subunit, that is, β_2_ or γ_2_). Moreover, the M2-M3 loop is also close to loop 2, enabling interaction of α_1_P277 with α_1_D54, α_1_H55, and α_1_M57 residues (pointing their functional groups in the direction of the TMD), and with the Cys-loop, it is mostly the residue L142. These residues form a kind of surface ensheating of α_1_P277 from above. In this region, the ECD-TMD interface is thus densely packed, making it possible that changes in the residue dimensions at position α_1_277 would affect molecular mechanisms underlying gating transitions. Consistent with this hypothesis, the α_1_P277A mutation, due to alanine size, which is most similar to that of native proline, would be expected to induce the smallest structural rearrangements and, therefore, weak changes in the receptor function. For other mutations, α_1_P277E, α_1_P277H, and α_1_P277K, the increase in side-chain size was bigger than in the case of alanine substitution that could explain more pronounced effects of these substitutions on the receptor kinetics. Moreover, we hypothesized that these point mutations, due to the close proximity of α_1_D54, α_1_H55, and α_1_M57 residues at loop 2, will favor electrostatic interactions especially for α_1_P277E and α_1_P277K cases. However, close proximity with residues with the opposite (α_1_P277K−α_1_D54) or same (α_1_P277E−α_1_D54) electrostatic charge turned out not to be a key factor in shaping GABA_A_R properties and resulted in a similar effect as seen in Tables 1–3. The reason for this observation may be that according to the experimental structures of the receptor,^59^ α_1_D54 forms a salt bridge with α_1_R220 located in the pre-M1 helix segment. The molecular effect of the charged mutations at α_1_P277 loci could be then just reduced to the disruption (or hindrance) of this interaction that is not dependent on the charge of the substituting residue. This scenario would be compatible with our hypothesis that steric interactions of the M2-M3 loop at the interface strongly contribute to the molecular mechanisms underlying gating transitions. Another argument for the importance of steric interactions is suggested by the analysis of GABA_A_R structures in distinct conformational states described in recent studies.^35−37,59^ As shown in Figure 8, transition between shut and desensitized states is associated with the movement of the M2-M3 loop and, therefore, of the α_1_P277 residue. Namely, upon transition from the desensitized state to shut state, the α_1_P277 residue moves toward the channel pore (1.9 and 1.5 A for respective two α subunits) and its distance to the H55 residue is reduced (by 1.0 and 1.2 A, respectively), indicating local structure tightening, thus affecting local steric interactions. Unfortunately, the open structure of GABA_A_R is not available and movements of considered residues remain unknown, but we may speculate that, considering the aforementioned tight packing of residues in this region, a similar scenario is likely to take place.

Altogether, we provide evidence that the P277 residue at the α_1_ subunit alters the GABA_A_R gating by changing the interaction of the M2-M3 loop with its surrounding in the interface region. Notably, mutation of this residue causes a minimal change in the binding kinetics pointing to the receptors’ feature that binding at the orthosteric binding site is a local phenomenon, whereas gating is a global one. It can be expected that better understanding of molecular mechanisms underlying GABA_A_R activation will provide a means to design clinically relevant drugs in the treatment of a number of diseases in which synaptic inhibition is impaired.

## Materials and Methods

### Cell Culture and Transfection

All of the electrophysiological experiments were performed with use of the HEK293 cell line (human embryonic kidney) from EACC (European Collection of Authenticated Cell Culture). The cells were cultured in Gibco DMEM with Glutamax supplemented with 10% FBS and 1% penicillin/streptomycin (all from Thermo Fisher Scientific) in a humidified atmosphere with 5% CO_2_ at 37 °C. For experiments, cells were replated on poly-d-lysine (1 μg/mL, Sigma)-coated 12 mm ø glass coverslips. Cells were allowed to grow on coverslips for at least 48 h. After that, transfection of prepared cells was done with FuGene HD (Promega, US) not earlier than 24 h before the experiment. The cDNA plasmid used for transfection was based on the coding sequence for rat (*R. norvegicus*) GABA_A_ receptor α_1_, β_2_, and γ_2L_ subunits, and also to help identify transfected cells, the eGFP plasmid was used. To ensure optimal expression of GABA_A_R and GFP plasmids for α_1_:β_2_:γ_2L_:eGFP, the following respective amount was used: 0.5:0.5:1.5:0.5 μg. Successfully transfected cells during measurements were visualized with a fluorescence illuminator (470 nm wavelength, CoolLED, UK) attached to a modular inverted microscope (Leica DMi8, Germany).

### Macroscopic Current Recordings

Macroscopic current recordings were performed 24 h after transfection using the patch-clamp technique. Kinetic description of macroscopic currents for WT, P277A, P277H, and P277K was assessed by outside-out excised-patch technique configuration. In the case of P277E for the dose–response relationship, it was obtained by recordings from lifted cell configuration. In each case, recordings were performed at a holding potential of −40 mV. Evoked macroscopic currents were filtered with an 8-pole low-pass Bessel filter set at 10 kHz using an Axopatch 200B (Molecular Devices, US) amplifier. The signal was then digitized with a Digidata 1550A card (Molecular Devices, US). Signals were acquired and stored with pClamp 10.7 software (Molecular Devices, US). Pipettes used in experiments were pulled from borosilicate glass (outer ø, 1.5 mm; inner ø, 1.05 mm; Science Products) using a P-97 horizontal puller (Sutter Instruments, US) to achieve the final resistance in the range of 3 ± 0.5 MΩ when filled with an intracellular Ringer solution that contained 137 mM KCl, 1 mM CaCl_2_, 2 mM ATP-Mg, 2 mM MgCl_2_, 10 mM K-gluconate, 11 mM EGTA, and 10 mM HEPES, with the pH adjusted to 7.2 with KOH. An external solution consisted of the following: 137 mM NaCl, 5 mM KCl, 2 mM CaCl_2_, 1 mM MgCl_2_, 10 mM HEPES, and 20 mM d-(+)-glucose (pH adjusted to 7.2 with NaOH). For experiments with high GABA concentrations (>10 mM, mainly for dose–response curve determination), a low-chloride solution was used to keep osmolarity at ∼330 mOsm: intrapipette solution: 87 mM KCl, 1 mM CaCl_2_, 2 mM MgCl_2_, 50 mM K-gluconate, 11 mM EGTA, 10 mM HEPES, and 2 mM ATP-Mg (pH adjusted to 7.2 with KOH); external solution: 87 mM NaCl, 5 mM KCl, 2 mM CaCl_2_, 1 mM MgCl_2_, 10 mM HEPES, and 20 mM d-(+)-glucose (pH adjusted to 7.2 with NaOH). Rapid application of GABA was performed with a theta glass tube (Science Products, Germany) mounted on a piezoelectric-driven translator (Physik Instrumente, Germany) as described in detail by refs (50), (60), and (61). Solutions were supplied into the two channels of the theta glass tube by a high-precision SP220IZ syringe pump (World Precision Instruments, US).

### Macroscopic Current Analysis

Dose–response relationships were described with standard Hill’s equation in the form:
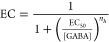
1where [GABA] is the agonist concentration, and *n_h_* is Hill’s coefficient. The time course of the macroscopic desensitization was fitted with a single exponential function (Clampfit, Molecular Devices, US) as:
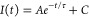
2

The current deactivation time course after application of saturating GABA pulse was fitted with a biexponential function:
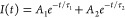
3

FR10 and FR500 parameters were calculated as:
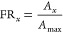
4where *A*_max_ refers to the current amplitude [pA], and *A_x_* refers to the current value [pA] after *x* ms after its peak.

The deactivation time constant was calculated as:
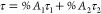
5where
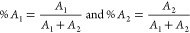
6

The rise time (RT) was calculated with the function build in Clampfit (Molecular Devices, US) as 10–90% of the macroscopic current onset.

#### Kinetic Modeling Based on Macroscopic Recordings

Kinetic modeling based on macroscopic measurements was done using ChannelLab software and in-house Python scripts. As the model scheme, the flipped Jones–Westbrook model was used in previous work.^50^ For the WT receptor, the initial rate values for optimization were taken from ref (9). Various initial rate values and conditions for mutant modeling were used: both unconstrained and constrained WT values and rate values taken from the single channel-based mutant models. Those multiple approaches were used to examine possible scenarios of valid rate sets. Because of the significant distance between α_1_P277 and the agonist binding site and low effect of mutations on the receptor EC_50_, the binding and unbinding rates were constrained to WT values in each case. For both WT and mutants, the rate optimization was done for the first ∼30 ms time window of the receptor response to the long pulse of GABA (excluding the slow desensitization period, not present in the model). Presented rate values are mean values for each fitted trace of the given receptor type.

Finally, the values of FR10 parameters were close to the steady state-to-peak ratio calculated as:
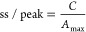
7where *A*_max_ refers to the current amplitude, and *C* was derived from eq 2.

### Single-Channel Recording

Single-channel recordings were performed in the cell-attached configuration of the patch-clamp technique at a holding pipette potential of 100 mV. The signal was filtered with an 8-pole low-pass Bessel filter set at 100 kHz using an Axopatch 200B (Molecular Devices, US) amplifier. The signal was digitized with a Digidata 1550B card (Molecular Devices, US) with the hum silencer option on. The acquisition of a signal was performed with pClamp 10.7 software (Molecular Devices, US). Pipettes used in experiments were pulled from borosilicate glass (outer ø, 1.5 mm; inner ø, 0.86 mm; Science Products, Germany) using a P-1000 horizontal puller (Sutter Instruments, US). Noise reduction was achieved by coating tips of pipettes with Sylgard (Dow Corning, US) and heat-polishing them, and the final pipette resistance was in the range of 10–15 MΩ. Ringer solution used for single-channel recording consisted of 102.7 mM NaCl, 20 mM Na-gluconate, 2 mM KCl, 2 mM CaCl_2_, 1.2 mM MgCl_2_, 10 mM HEPES (Carl Roth, Germany), 20 mM TEA-Cl, 14 mM d-(+)-glucose, and 15 mM sucrose (Carl Roth, Germany) and dissolved in deionized water with the pH adjusted to 7.4 with 2 M NaOH; for intrapipette solution, Ringer solution was supplemented with 10 mM GABA. To further reduce the impact of noise on the single-channel record quality, the amount of the extracellular solution was kept at a minimal possible level. Recorded traces were selected for further analysis if the patches had a stable seal resistance of at least 10 GΩ.

### Single-Channel Analysis

All stable patches at first were filtered to achieve a signal-to-noise ratio of around 15. The final cut-off frequency (*f*_c_) was calculated as:
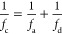
8

All mutations showed cluster activity with different modes of activity.^8,48,49^ For better distinction of different activity modes, all clusters were prescanned with pClamp with an event detection function to establish the *P*_open_ of each cluster. Selected clusters of dominant activity mode in the next step were idealized with SCAN software (DCProgs, http://www.onemol.org.uk/, kindly provided by David Colquhoun) and stored in .scn files. For further analysis, only traces containing ∼10,000 events (understood as a number of closures and openings summed up) were proceeded. In next step of analysis, .scn files were used to construct distribution of open and shut times with EKDIST (DCProgs). Determination of the rate constant between transition states of GABA_A_R was performed by Hjcfit (DCProgs) and stored in .scn files by applying the maximum likelihood method for the predefined kinetic scheme based on distribution of shut and open times. Since all recordings were performed under sat. [GABA] with the agonist constantly present in intrapipette solution, the binding step and their respectable kinetic rates were omitted in our modeling.^8,49^ The burst length and *P*_open_ were calculated with EKDIST (DCProgs) using the *t*-critical value, which is based on the Jackson criterion.^62^ All analysis steps are described further in ref (49).

## Author’s Contribution

P.T.K. performed experiments, data analysis, model simulations, alignment, and final data visualization and wrote the first draft of the manuscript. M.A.M. performed macroscopic recording simulation, visualization of simulated macroscopic data, and structure visualization and wrote the first draft of the manuscript. J.W.M. conceived the project, provided financial support, supervised project realization, and participated in designing of the experiments, data analysis, model simulations, and writing and editing of the final version of the manuscript.

## Abbreviations

ECD: extracellular domain
TMD: transmembrane domain
GABA_A_R: γ-aminobutyric acid receptor type A
[GABA]: γ-aminobutyric acid concentration
WT: wild type of γ-aminobutyric acid receptor type A
P277: 277th residue of the α_1_ subunit of γ-aminobutyric acid receptor type A
P277A/E/H/K: substitution of the P277 residue of the α_1_ subunit of γ-aminobutyric acid receptor type A with alanine, glutamic acid, histidine, and lysine
FR10/500: percentage of the remaining current evoked by [GABA] application at 10/500 ms after peak amplitude
s-ch: single-channel
